# Supplementary material for: Association of trends in child undernutrition and implementation of the National Rural Health Mission in India: A nationally representative serial cross-sectional study on data from 1992 to 2015
Source: PLoS Med. 2022 Apr 8;19(4):e1003957. doi: 10.1371/journal.pmed.1003957 (PMC9032440; doi:10.1371/journal.pmed.1003957)
Supplement: S1 Text — Table A: Weighted distribution of eligible children under the age of 3 measured for undernutrition in Indian NFHS from 1992 to 2015. Table B: Weighted distribution of ineligible children under the age of 3 measured for undernutrition in Indian NFHS from 1992 to 2015. Table C: Estimates of child undernutrition indicators of eligible children under the age of 3 measured for undernutrition in Indian NFHS from 1992 to 2015. NFHS, National Family Health Survey. (DOCX) [file pmed.1003957.s001.docx]

| **Supplementary Table A: Weighted distribution of eligible children under the age of 3 measured for undernutrition in Indian National Family Health Surveys from 1992 to 2015** | | | | | | | | | | | | | | | | |
| --- | --- | --- | --- | --- | --- | --- | --- | --- | --- | --- | --- | --- | --- | --- | --- | --- |
|  | **All States** | | | | **Normal Focus** | | | | **High Focus** | | | | **Northeast Focus** | | | |
|  | **1992** | **1998** | **2005** | **2015** | **1992** | **1998** | **2005** | **2015** | **1992** | **1998** | **2005** | **2015** | **1992** | **1998** | **2005** | **2015** |
| **Number of Children** | 18,396 | 22,390 | 22,442 | 124,224 | 7,504 | 8,607 | 8,025 | 29,982 | 8,335 | 10,530 | 9,990 | 75,799 | 2,557 | 3,253 | 4,427 | 18,443 |
| **Wealth Quintile** |  |  |  |  |  |  |  |  |  |  |  |  |  |  |  |  |
| 1 (Poorest) | 21.7 | 21.8 | 25.3 | 24.8 | 8.3 | 15.0 | 14.2 | 8.9 | 30.6 | 29.1 | 33.5 | 36.8 | 25.2 | 20.4 | 21.1 | 25.1 |
| 2 | 21.0 | 21.7 | 22.2 | 22.1 | 10.1 | 17.8 | 17.6 | 18.1 | 27.4 | 25.1 | 24.7 | 24.0 | 31.0 | 29.0 | 33.5 | 38.4 |
| 3 | 18.9 | 20.4 | 19.7 | 20.2 | 21.2 | 20.8 | 21.2 | 24.8 | 17.1 | 19.5 | 18.2 | 16.6 | 21.8 | 26.2 | 24.2 | 19.8 |
| 4 | 21.2 | 19.9 | 18.6 | 18.4 | 32.3 | 24.2 | 25.1 | 26.5 | 14.4 | 15.4 | 14.3 | 12.7 | 13.8 | 17.8 | 13.7 | 11.7 |
| 5 (Richest) | 17.2 | 16.3 | 14.3 | 14.6 | 28.1 | 22.1 | 21.8 | 21.7 | 10.6 | 10.9 | 9.4 | 9.9 | 8.2 | 6.7 | 7.5 | 5.1 |
| **Mother Education** |  |  |  |  |  |  |  |  |  |  |  |  |  |  |  |  |
| None (0 years) | 63.8 | 52.8 | 48.7 | 28.5 | 46.4 | 39.0 | 30.8 | 14.0 | 76.5 | 67.7 | 62.3 | 40.2 | 56.2 | 45.3 | 35.5 | 19.3 |
| Primary (1-5 years) | 11.3 | 15.8 | 13.7 | 13.4 | 15.0 | 18.5 | 14.9 | 11.9 | 8.2 | 12.6 | 12.3 | 14.4 | 16.9 | 21.1 | 20.5 | 17.1 |
| Secondary+ (>5 years) | 24.9 | 31.4 | 37.7 | 58.1 | 38.6 | 42.4 | 54.3 | 74.1 | 15.3 | 19.7 | 25.4 | 45.4 | 26.9 | 33.6 | 44.0 | 63.6 |
| **Rural** | 76.8 | 76.2 | 75.6 | 72.5 | 64.4 | 68.4 | 64.8 | 59.6 | 84.2 | 83.4 | 82.4 | 81.5 | 87.0 | 88.5 | 86.6 | 85.8 |
| **Female** | 49.5 | 48.2 | 47.7 | 48.2 | 49.4 | 48.1 | 47.2 | 48.5 | 49.6 | 48.3 | 48.0 | 48.0 | 51.0 | 48.9 | 49.2 | 48.7 |
| **1st Born** | 24.1 | 27.3 | 29.1 | 37.3 | 28.6 | 32.9 | 36.3 | 43.7 | 21.2 | 21.4 | 23.6 | 32.2 | 23.5 | 26.9 | 33.7 | 40.8 |
| **Hindu Religion** | 77.4 | 79.4 | 78.3 | 78.6 | 73.1 | 76.3 | 74.3 | 76.0 | 82.7 | 84.7 | 83.4 | 82.7 | 54.4 | 47.0 | 50.9 | 46.9 |
| **Mean Child Age in Months (SE)** | 17.1 (0.09) | 17.4 (0.08) | 18.1 (0.08) | 18.2 (0.04) | 17.5 (0.11) | 17.7 (0.12) | 18.7 (0.12) | 18.5 (0.07) | 16.7 (0.13) | 17.1 (0.10) | 17.8 (0.11) | 18.0 (0.04) | 17.5 (0.28) | 17.0 (0.27) | 17.8 (0.30) | 18.2 (0.11) |
| **Mean Birth Order**  **(SE)** | 3.1 (0.02) | 2.8 (0.02) | 2.8 (0.02) | 2.2 (0.01) | 2.7 (0.03) | 2.4 (0.02) | 2.2 (0.03) | 1.8 (0.01) | 3.4 (0.03) | 3.3 (0.03) | 3.2 (0.03) | 2.5 (0.01) | 3.4 (0.08) | 3.0 (0.05) | 2.7 (0.08) | 2.2 (0.02) |
| **Mean Age at First Marriage (SE)** | 16.9 (0.03) | 17.0 (0.04) | 17.2 (0.04) | 19.5 (0.04) | 17.4 (0.06) | 17.5 (0.06) | 17.9 (0.06) | 20.0 (0.07) | 16.5 (0.05) | 16.4 (0.04) | 16.6 (0.05) | 19.1 (0.04) | 17.6 (0.15) | 18.2 (0.15) | 18.4 (0.20) | 20.8 (0.12) |
| **Mean Maternal Age (SE)** | 26.0 (0.06) | 25.2 (0.05) | 25.6 (0.06) | 26.0 (0.02) | 24.9 (0.10) | 24.5 (0.07) | 24.9 (0.07) | 25.4 (0.04) | 26.7 (0.08) | 25.9 (0.07) | 26.0 (0.08) | 26.4 (0.03) | 26.0 (0.22) | 26.1 (0.16) | 26.3 (0.20) | 26.6 (0.07) |
| a: Composite Index of Anthropometric Failure (Underweight or Stunted or Wasted); b: other backward class was considered as general in 1992 | | | | | | | | | | | | | | | | |

| **Supplementary Table B: Weighted distribution of ineligible children under the age of 3 measured for undernutrition in Indian National Family Health Surveys from 1992 to 2015** | | | | | | | | | | | | | | | | |
| --- | --- | --- | --- | --- | --- | --- | --- | --- | --- | --- | --- | --- | --- | --- | --- | --- |
|  | **All States** | | | | **Normal Focus** | | | | **High Focus** | | | | **Northeast Focus** | | | |
|  | **1992** | **1998** | **2005** | **2015** | **1992** | **1998** | **2005** | **2015** | **1992** | **1998** | **2005** | **2015** | **1992** | **1998** | **2005** | **2015** |
| **Number of Children** | 30,563 | 10,636 | 29,113 | 135,403 | 12,699 | 3,247 | 10,986 | 34,255 | 15,062 | 6,148 | 12,899 | 82,424 | 2,802 | 1,241 | 5,228 | 18,724 |
| **Wealth Quintile** |  |  |  |  |  |  |  |  |  |  |  |  |  |  |  |  |
| 1 (Poorest) | 22.6 | 26.0 | 25.6 | 25.9 | 16.4 | 15.2 | 14.7 | 9.4 | 28.4 | 32.5 | 33.8 | 39.2 | 29.5 | 20.7 | 22.9 | 27.5 |
| 2 | 22.7 | 23.8 | 22.6 | 21.9 | 19.4 | 18.1 | 17.8 | 18.3 | 25.4 | 26.8 | 25.5 | 23.8 | 31.9 | 29.4 | 31.5 | 37.8 |
| 3 | 21.7 | 19.8 | 19.9 | 19.5 | 21.9 | 21.0 | 22.7 | 24.1 | 21.7 | 18.8 | 17.7 | 15.9 | 19.1 | 25.6 | 23.5 | 19.4 |
| 4 | 18.7 | 17.9 | 17.6 | 17.9 | 23.2 | 26.0 | 23.1 | 25.9 | 14.6 | 13.2 | 13.8 | 11.9 | 12.9 | 16.4 | 13.9 | 10.6 |
| 5 (Richest) | 14.4 | 12.5 | 14.3 | 14.8 | 19.0 | 19.6 | 21.7 | 22.3 | 9.9 | 8.7 | 9.3 | 9.3 | 6.6 | 8.0 | 8.3 | 4.7 |
| **Mother Education** |  |  |  |  |  |  |  |  |  |  |  |  |  |  |  |  |
| None (0 years) | 65.7 | 59.2 | 51.3 | 31.7 | 54.3 | 38.5 | 33.9 | 16.1 | 77.7 | 72.2 | 65.0 | 44.9 | 59.1 | 43.2 | 37.2 | 24.7 |
| Primary (1-5 years) | 12.7 | 13.6 | 14.1 | 14.3 | 16.5 | 18.0 | 15.9 | 13.2 | 8.4 | 10.7 | 12.1 | 15.0 | 19.2 | 20.7 | 22.9 | 18.4 |
| Secondary+ (>5 years) | 21.6 | 21.2 | 34.7 | 54.0 | 29.2 | 43.5 | 50.2 | 70.8 | 14.0 | 17.1 | 22.9 | 40.1 | 21.7 | 36.1 | 39.9 | 56.9 |
| **Rural** | 77.6 | 81.0 | 74.0 | 71.4 | 70.8 | 71.0 | 62.9 | 57.9 | 83.5 | 86.1 | 81.4 | 81.6 | 89.2 | 90.6 | 84.1 | 85.1 |
| **Female** | 48.6 | 47.9 | 48.0 | 47.4 | 49.1 | 47.9 | 46.9 | 47.1 | 48.0 | 48.1 | 48.6 | 47.6 | 50.0 | 44.1 | 50.4 | 47.8 |
| **1st Born** | 29.3 | 32.2 | 31.2 | 39.8 | 32.5 | 41.1 | 38.5 | 46.6 | 26.5 | 26.8 | 26.0 | 34.1 | 22.4 | 34.6 | 30.4 | 41.1 |
| **Hindu Religion** | 80.6 | 79.1 | 78.3 | 78.8 | 77.0 | 77.4 | 76.6 | 76.4 | 86.1 | 81.8 | 81.4 | 82.8 | 53.2 | 52.8 | 50.3 | 45.6 |
| **Mean Child Age in Months (SE)** | 35.2 (0.20) | 51.2 (0.64) | 47.0 (0.20) | 47.4 (0.09) | 32.5 (0.22) | 37.4 (0.73) | 44.8 (0.30) | 45.3 (0.17) | 37.5 (0.34) | 59.7 (0.89) | 48.5 (0.28) | 49.2 (0.10) | 40.8 (0.90) | 42.9 (2.1) | 48.0 (0.76) | 47.9 (0.28) |
| **Mean Birth Order**  **(SE)** | 3.1 (0.02) | 2.8 (0.02) | 2.8 (0.02) | 2.2 (0.01) | 2.6 (0.03) | 2.2 (0.04) | 2.2 (0.02) | 1.8 (0.01) | 3.2 (0.02) | 3.2 (0.04) | 3.2 (0.03) | 2.5 (0.01) | 3.4 (0.09) | 2.7 (0.08) | 2.8 (0.08) | 2.3 (0.02) |
| **Mean Age at First Marriage (SE)** | 16.5 (0.03) | 16.7 (0.05) | 17.1 (0.04) | 19.7 (0.04) | 16.7 (0.05) | 17.4 (0.08) | 17.7 (0.06) | 20.4 (0.08) | 16.2 (0.04) | 16.1 (0.05) | 16.6 (0.05) | 19.1 (0.04) | 17.5 (0.17) | 18.0 (0.21) | 18.2 (0.17) | 21.2 (0.15) |
| **Mean Maternal Age (SE)** | 25.7 (0.05) | 24.6 (0.07) | 26.8 (0.05) | 27.5 (0.02) | 24.9 (0.07) | 23.6 (0.09) | 26.1 (0.07) | 27.0 (0.04) | 26.4 (0.07) | 25.1 (0.09) | 27.3 (0.08) | 28.0 (0.03) | 26.6 (0.20) | 25.3 (0.27) | 27.9 (0.19) | 28.4 (0.08) |
| a: Composite Index of Anthropometric Failure (Underweight or Stunted or Wasted); b: other backward class was considered as general in 1992 | | | | | | | | | | | | | | | | |

| **Supplementary Table C: Estimates of child undernutrition indicators of eligible children under the age of 3 measured for undernutrition in Indian National Family Health Surveys from 1992 to 2015** | | | | | | | | | | | | | | | | |
| --- | --- | --- | --- | --- | --- | --- | --- | --- | --- | --- | --- | --- | --- | --- | --- | --- |
|  | **All States** | | | | **Normal Focus** | | | | **High Focus** | | | | **Northeast Focus** | | | |
|  | **1992** | **1998** | **2005** | **2015** | **1992** | **1998** | **2005** | **2015** | **1992** | **1998** | **2005** | **2015** | **1992** | **1998** | **2005** | **2015** |
| **Number of Children** | 18,396 | 22,390 | 22,442 | 124,224 | 7,504 | 8,607 | 8,025 | 29,982 | 8,335 | 10,530 | 9,990 | 75,799 | 2,557 | 3,253 | 4,427 | 18,443 |
| **Unadjusted** |  |  |  |  |  |  |  |  |  |  |  |  |  |  |  |  |
| Stunted (Chronic) | 57.0 | 52.9 | 46.5 | 36.9 | 50.3 | 46.6 | 42.2 | 31.3 | 62.1 | 61.2 | 50.4 | 41.8 | 56.6 | 48.8 | 40.8 | 32.7 |
| Wasted (Acute) | 28.5 | 21.6 | 26.1 | 26.6 | 25.3 | 19.8 | 20.7 | 25.1 | 32.2 | 28.5 | 31.4 | 28.3 | 17.6 | 18.2 | 20.8 | 19.6 |
| CIAF (Overall) | 73.0 | 65.1 | 64.4 | 58.5 | 66.6 | 60.4 | 58.2 | 52.6 | 77.8 | 75.3 | 69.5 | 63.5 | 67.9 | 65.7 | 56.9 | 50.1 |
| Underweight | 47.4 | 43.9 | 40.5 | 33.6 | 41.4 | 37.9 | 33.5 | 28.7 | 52.8 | 49.7 | 46.9 | 38.4 | 40.7 | 30.3 | 34.0 | 25.1 |
| Stunted and Wasted | 11.9 | 9.6 | 9.4 | 6.4 | 9.7 | 8.3 | 6.7 | 5.4 | 14.4 | 11.5 | 12.4 | 7.6 | 7.8 | 4.1 | 6.6 | 4.0 |
| **Model A** |  |  |  |  |  |  |  |  |  |  |  |  |  |  |  |  |
| Stunted (Chronic) | 55.6 | 54.1 | 46.5 | 36.9 | 49.1 | 47.5 | 42.0 | 31.6 | 60.7 | 59.6 | 50.4 | 41.9 | 55.6 | 48.7 | 40.7 | 32.7 |
| Wasted (Acute) | 27.6 | 22.9 | 26.1 | 26.7 | 24.5 | 19.9 | 20.7 | 25.3 | 31.1 | 25.8 | 31.4 | 28.4 | 17.2 | 19.1 | 20.9 | 19.7 |
| CIAF (Overall) | 73.0 | 68.4 | 64.2 | 58.6 | 65.0 | 61.0 | 57.9 | 52.9 | 76.4 | 74.0 | 69.3 | 63.5 | 66.6 | 64.6 | 56.7 | 50.0 |
| Underweight | 52.8 | 48.3 | 45.1 | 37.3 | 45.7 | 41.0 | 37.0 | 31.0 | 58.9 | 55.3 | 52.2 | 43.3 | 46.1 | 33.5 | 37.9 | 27.8 |
| Stunted and Wasted | 14.4 | 11.2 | 11.0 | 7.5 | 11.1 | 9.3 | 7.7 | 6.0 | 17.7 | 13.8 | 14.8 | 9.1 | 9.5 | 4.5 | 7.9 | 3.9 |
| CIAF: Composite Index of Anthropometric Failure (stunted, wasted, or underweight)  Model A adjusted for month of survey as well as religion and caste of the child's household | | | | | | | | | | | | | | | | |
